# Backtracking behavior in viral RNA-dependent RNA polymerase provides the basis for a second initiation site

**DOI:** 10.1093/nar/gkv1098

**Published:** 2015-10-22

**Authors:** David Dulin, Igor D. Vilfan, Bojk A. Berghuis, Minna M. Poranen, Martin Depken, Nynke H. Dekker

**Affiliations:** 1 Department of Bionanoscience, Kavli Institute of Nanoscience Delft, Delft University of Technology, Lorentzweg 1, 2628 CJ Delft, The Netherlands; 2 Department of Biosciences, University of Helsinki, Viikki Biocenter 1, P.O. Box 56 (Viikinkaari 9), 00014 Helsinki, Finland

## Abstract

Transcription in RNA viruses is highly dynamic, with a variety of pauses interrupting nucleotide addition by RNA-dependent RNA polymerase (RdRp). For example, rare but lengthy pauses (>20 s) have been linked to backtracking for viral single-subunit RdRps. However, while such backtracking has been well characterized for multi-subunit RNA polymerases (RNAPs) from bacteria and yeast, little is known about the details of viral RdRp backtracking and its biological roles. Using high-throughput magnetic tweezers, we quantify the backtracking by RdRp from the double-stranded (ds) RNA bacteriophage Φ6, a model system for RdRps. We characterize the probability of entering long backtracks as a function of force and propose a model in which the bias toward backtracking is determined by the base paring at the dsRNA fork. We further discover that extensive backtracking provides access to a new 3′-end that allows for the *de novo* initiation of a second RdRp. This previously unidentified behavior provides a new mechanism for rapid RNA synthesis using coupled RdRps and hints at a possible regulatory pathway for gene expression during viral RNA transcription.

## INTRODUCTION

Organisms in all domains of life share a need to replicate and transcribe their genome (1). Replication and transcription are highly dynamic processes in which rapid nucleotide incorporation is frequently interrupted by a variety of pauses that differ in nature depending on the molecular machinery involved (2–4). For example, the T7 replisome pauses during primer synthesis (5), *Escherichia coli* RNAP pauses in a sequence-dependent manner during elongation (6,7), and the incorporation of incorrect nucleotides causes the dsRNA bacteriophage Φ6 RdRp to pause (8). In both eukaryotic and bacterial multi-subunit RNAPs, long pauses (>10 s) have been assigned to backtracked pauses, where the RNAP diffuses upstream on the DNA template (9,10). During a backtracked pause, the nascent RNA and the template are held in register, and the nascent 3′-end exits the core RNAP through the NTP channel. Backtracking ends either through spontaneous realignment of the 3′-end and the catalytic site, or through the creation of a new 3′-end at the catalytic site via protein-dependent cleavage (9–12). As the backtracked state is a stochastic and rare event, it is particularly well suited to study by single-molecule approaches that avoid the inherent averaging of bulk experiments. Though backtracking and its attendant dynamics have been studied in bacteria and eukaryotes using single-molecule force spectroscopy (9,10), the phenomenon has only recently been observed for an RdRp of viral origin (8).

RdRps are responsible for both replication and transcription in RNA viruses, which remain at the forefront of many human and animal epidemics. The genomes of RNA viruses may be single-stranded with either sense ((+)RNA) (e.g. poliovirus, hepatitis C virus) or anti-sense ((−)RNA) (e.g. influenza virus, ebola virus), or they can be double-stranded (e.g. rotavirus, bacteriophage Φ6). Due to the high error rate of their RdRps (13), RNA viruses evolve very rapidly (14,15) making it challenging to design effective vaccines. RdRps also play a central role in viral RNA recombination—a separate pathway for viral evolution (16). RdRps of RNA viruses share a conserved catalytic site that is also found among the reverse transcriptases (e.g. human immunodeficiency virus, HIV) (17–19). Given the relative inefficiency of vaccines, RdRps have become direct targets for drug development. For example, ribavirin, a mutagen nucleotide analogue that is incorporated by RdRp, has been introduced as therapy for hepatitis C and poliovirus infections (20).

We present a study of P2, the RdRp of the bacteriophage Φ6 (21) and a general model for RNA virus RdRps. P2 initiates transcription and replication *de novo* on a free 3′ RNA end (22–25). Following initiation, P2 enters the RNA elongation phase, which we have recently characterized at the single-molecule level using a highly parallelized magnetic tweezers assay. This approach, which is particularly powerful in studying rare events, has revealed evidence for a previously unknown catalytically competent and error-prone pathway (8). Presently, we utilize the power of this approach to study RdRp backtracking. We demonstrate that the probability for the polymerase to enter long backtracks state is force dependent, and we provide a mechanochemical model explaining this force dependence in terms of re-hybridization of the RNA strands at the fork. Interestingly, following long backtracks we occasionally observe a stable and processive reversal of the direction of transcription. We propose that the 3′ RNA that protrudes as a result of backtracking provides a new initiation site for a second P2. Following successful initiation, such a second P2 displaces the first P2 in the upstream direction along its template. Strikingly, at low tether forces the apparent nucleotide addition rate of the second P2 RdRp is twice faster than that of the first, highlighting a possible pathway for rapid RNA synthesis during viral transcription. Given the structural similarity amongst different viral RdRps (18), it is likely that such a mechanism would be conserved amongst the viral RdRps that rely on *de novo* initiation (e.g. hepatitis C virus)(26).

## MATERIALS AND METHODS

### Magnetic tweezers apparatus

The magnetic tweezers assay and the flow-cell preparation used here have been previously described in (8,27,28).

### dsRNA construct

The construct employed here is made of four single-stranded (ss) RNA strands annealed to one complementary strand of 4 kb in length as described in detail previously (8). To link the construct to the bead, a ssRNA strand containing biotins is annealed at one end of the complementary strand. To attach the construct to the surface, the other end of the complementary strand is annealed to a ssRNA strand containing digoxygenin. The template strand, ≈2.9 kb in length, contains a free 3′-end starting with 3 C residues followed by 15 U residues. A ssRNA strand is annealed as a spacer between the biotinylated strand and the template strand.

### Reaction buffer for P2

The reaction buffer is composed of 50 mM HEPES pH 7.9, 20 mM ammonium acetate, 3% w/v polyethylene glycol 4000, 0.1 mM EDTA (pH 8.0), 5 mM MgCl_2_, 2 mM MnCl_2_, 0.01% Triton X-100, 5% Superase RNase inhibitor (Life Technologies), 20 μg/ml bovine serum albumin, and an optimal concentration of nucleotides, [NTP]_opt_, of 1 mM ATP and GTP, 0.2 mM CTP and UTP, coming from HPLC-purified stock solutions. This concentration has been shown to be optimal for initiation of the RdRp on the 3′-end of an RNA template starting with 3 C residues followed by 15 U residues (8,29,30).

### Single-molecule transcription experiments

Once the RNA construct is calibrated inside a flow cell containing P2 reaction buffer (8), 9 nM of P2 is flushed in with the reaction buffer. We perform experiments at 21°C for 1 h at constant force and fixed NTP concentration while recording images of the magnetic beads at 25 Hz. Subsequently, the images are analyzed in real time using custom-written routines in Labview and CUDA nVidia to determine their (*x*, *y*, *z*) position (27). Up to 800 beads can be followed in real time at 25 Hz with this assay. Distinct traces are low-pass filtered at 0.5 Hz and synchronized. The changes in tether extension are converted into numbers of transcribed nucleotides using the force-extension relationships for dsRNA and ssRNA constructs obtained in P2 reaction buffer (8), as previously described (31).

### Generation of dwell-time distributions

**Figure 1.**
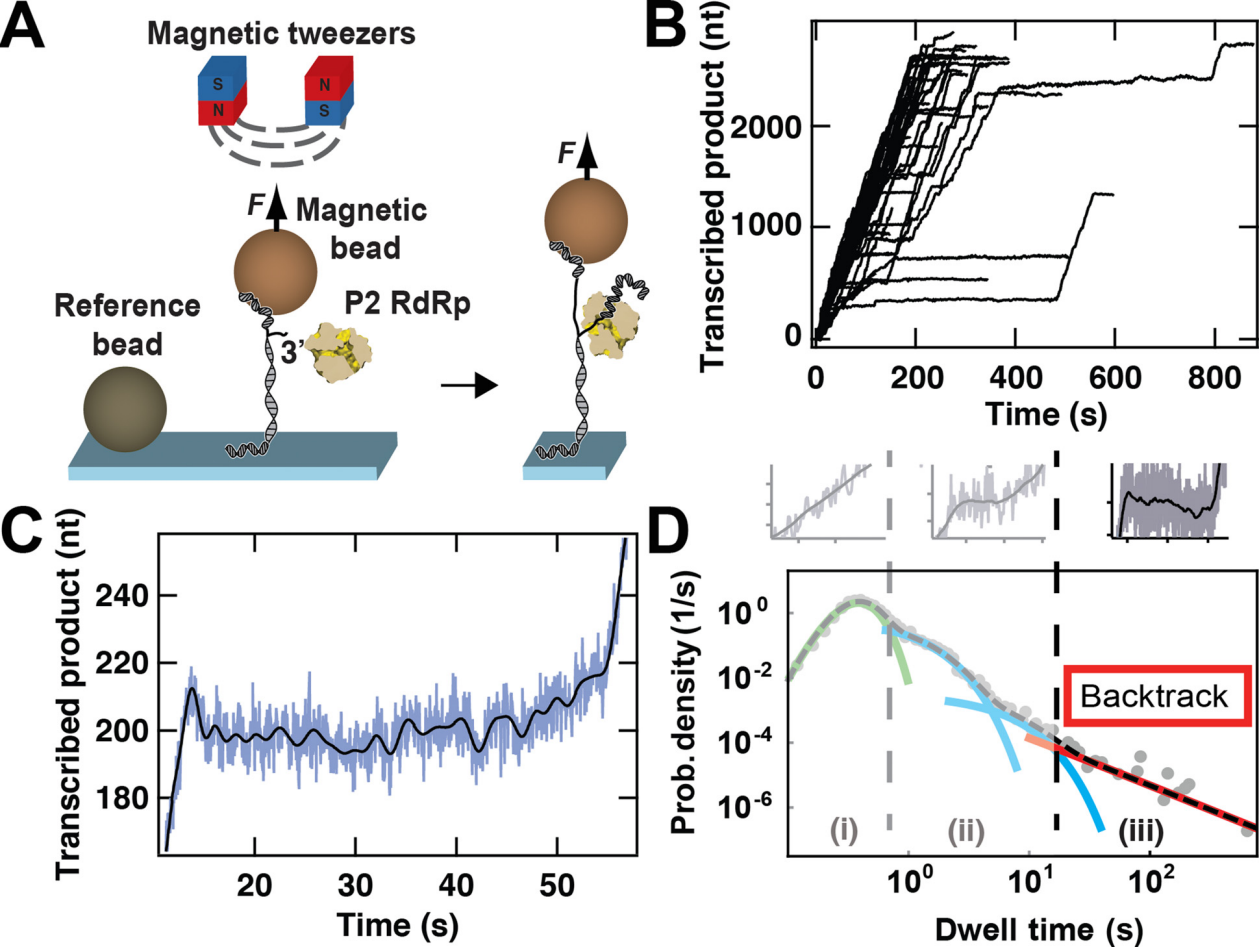
Experimental configuration of the magnetic tweezers assay and identification of RdRp backtracking. **(A)** Schematic representation of the experimental assay (not to scale). A predominantly dsRNA construct, attached to the surface by one end and to a micron-sized magnetic bead by the other end, experiences a constant force applied to a magnetic bead (light brown sphere) via a pair of permanent magnets (red and blue cubes) placed above the flow cell (not shown). One of the strands of the construct has a free 3′ end for initiation by a single P2 RdRp. A reference bead (dark brown sphere) is non-specifically adsorbed to the surface to correct for the influence of mechanical drift. During elongation, P2 RdRp synthesizes a complementary copy to the template strand, and in doing so exposes a ssRNA strand between the flow cell surface and the magnetic bead. The resulting change in extension is monitored and converted into a number of incorporated nucleotides. **(B)** Transcription activity by P2 RdRp versus time monitored using parallelized detection, yielding 52 traces in a single experiment. The experimental conditions include an applied force of 30 pN, an acquisition frequency of 25 Hz (low-pass filtered at 0.5 Hz) and 1 mM ATP/GTP and 0.2 mM CTP/UTP. **(C)** A sample trace illustrating backtracking behavior by P2 RdRp. A decrease in the length of the transcribed product by ≈15 nts is observed at ≈15 s. Subsequently, P2 RdRp pauses for ≈40 s before resuming elongation. For this trace, the applied force is 35 pN (Materials and Methods). The raw data (blue) are acquired at 25 Hz and filtered at 0.5 Hz (black line). **(D)** A dwell-time distribution extracted from 52 traces of P2 transcription activity acquired at 20 pN and [NTP]_opt_ (gray dots). We fit this distribution to a stochastic-pausing model by using MLE (dashed black line)(8). For clarity, we individually plot each contribution to the dwell-time distribution: in green, a Gamma distribution capturing the elongation peak; in blue, the first short exponential pause (Pause 1) and the second short exponential pause (Pause 2); and in red, the power law distribution of pause times originating from backtracking. Above the dwell-time distribution, representative P2 activity events are plotted: from left to right, fast incorporation without pause (light gray), short pauses (light gray) and long backtracked pauses (dark gray). The fit parameters for the Gamma distribution and the exponential distributions extracted from the MLE are available in (8).

We use a dwell-time analysis (8) to analyze the low-pass filtered data obtained as described above (Figure 1C, inset). Thus, we scan each transcription trace with a 10 nt transcription window, and for each such window we record the time needed for the polymerase to transcribe through it, and record this as the dwell-time. We then slide the transcription window forward by 10 nt and record the subsequent dwell-time. This procedure is repeated until the end of the trace is reached, and the dwell-times that result from an individual trace are binned with those resulting from identical conditions (e.g., same force) to yield a dwell-time distribution. Looking at dwell-time distribution for individual polymerases, it is clear that a small percentage spend a much longer-than-average time pausing. To prevent these pause-prone polymerases from biasing the data, we remove the 5% with the most extreme pause densities.

## RESULTS

### Observation of Φ6 P2 RdRp elongation kinetics using a highly parallelized magnetic tweezers

To observe the activity of Φ6 P2 RdRp during the elongation phase, we use our previously described assay (8). Briefly, a dsRNA construct is tethered to a magnetic bead and kept at a constant tension applied through a pair of permanent magnets positioned above the flow chamber. The dsRNA construct has one strand partially non-hybridized at its 3′-end, hence providing a template for P2 initiation. Once a P2 RdRp has initiated, it displaces the template strand from the complementary strand that tethers the bead to the surface (Figure 1A). Because of the length difference between ssRNA and dsRNA, we can monitor the motion of each elongating P2 with a resolution ≈5 nt at 0.5 Hz (8). The massive parallelization of our instrument and computational approach provides the possibility of real-time acquisition of up to 52 polymerase activity traces simultaneously (Figure 1B) (27).

When the RdRp backtracks, the template strand rehybridizes to the tether strand, resulting in a shortening of the construct, hence in an apparent backward motion of the polymerase (Figure 1C). When we provide an applied force as high as 30–35 pN, the spatiotemporal resolution of our apparatus is sufficient to observe backward motion of 10 nt or longer. In Figure 1C, we show how pauses of ≈40 s follow an initial backward motion of ≈10 bases. Due to a decreased spatiotemporal resolution at lower forces, we cannot directly observe the backward motion of P2 for most of the long pauses (>20 s), but instead infer their existence by their duration, knowing that the shorter pauses (1–10 s) derive from a different origin (8).

We performed a dwell-time analysis for each experimental condition by measuring the time it takes the polymerase to transcribe through consecutive windows of 10 nt along the trace (8). The obtained dwell-time distribution contains the features of different probability distribution functions (Figure 1D, gray dots). We determined three different trends in the dwell-time distribution: a short-time gamma distributed peak ((i) in Figure 1D) originating from the P2 polymerase incorporating 10 successive nt without a pause, an exponential shoulder situated at the intermediate times ((ii) in Figure 1D), and a long-lived pause with a broad distribution ((iii) in Figure 1D) consistent with an algebraic decay generated by backtracking pauses (8,10,32,33). The parameters of each distribution can be extracted via Maximum Likelihood Estimation (MLE) (black dashed line in Figure 1D) (8).

### The nature of the P2 RdRp backtracked state is force dependent

**Figure 2.**
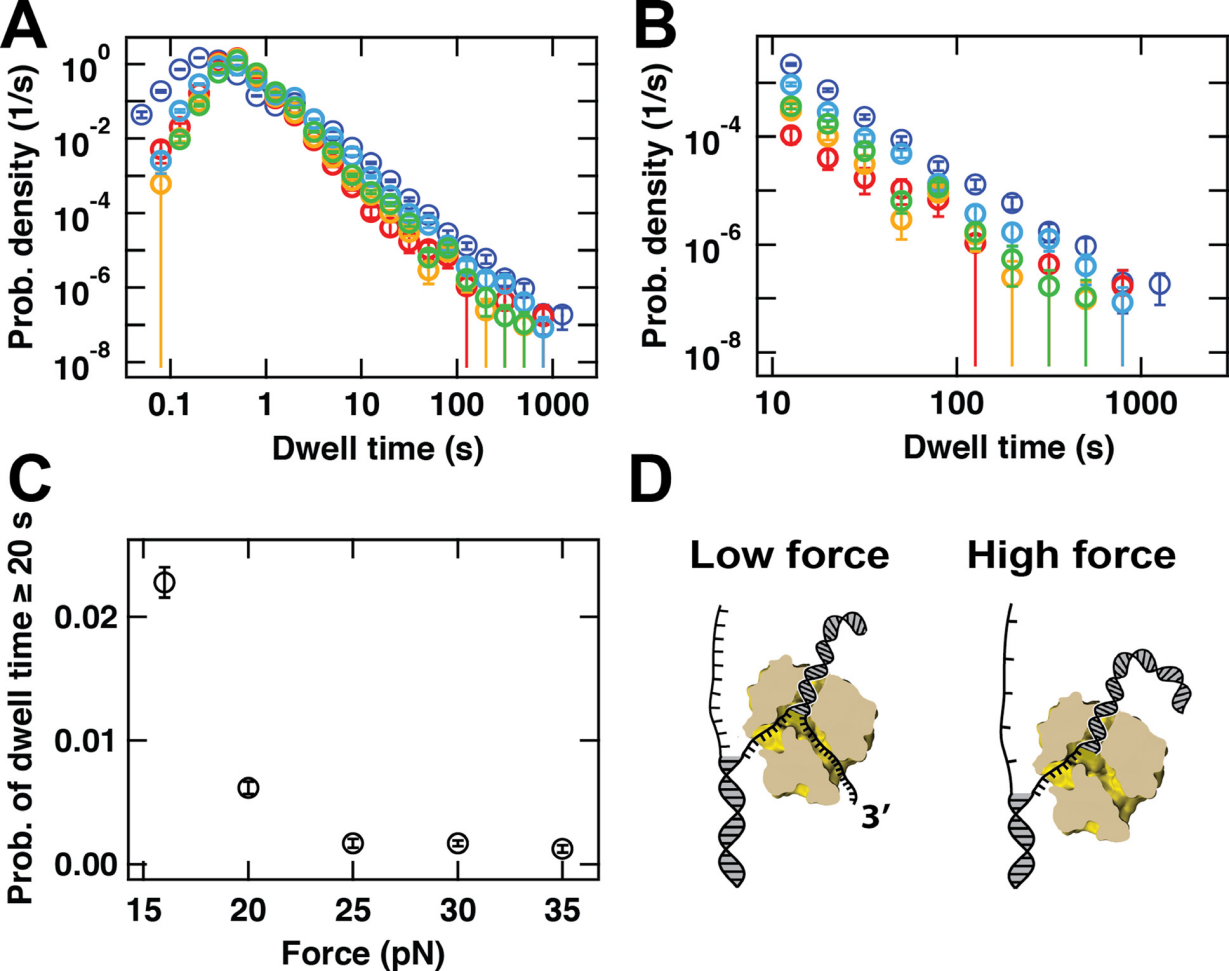
Force dependence of the probability of entering into a backtracked state for P2 RdRp. **(A)** Probability density distributions for P2 RdRp transcriptional activity acquired at 16 pN (dark blue, 102 traces), 20 pN (light blue, 184 traces), 25 pN (green, 200 traces), 30 pN (yellow, 210 traces) and 35 pN (red, 76 traces). The error bars correspond to one standard deviation estimated from 1000 bootstraps. **(B)** A zoom-in of (A) for dwell-times longer than 10 s. **(C)** Probability that a dwell-time exceeds 20 s as a function of the applied force. The error bars are the standard deviation of the distribution extracted from 1000 bootstraps. **(D)** Proposed model that accounts for the force-dependence of the probability of finding P2 RdRp in a backtracked state. At low force (small distance between single nucleotides on the non-template strand), the tension at the dsRNA fork is small enough to allow the rehybridization of the template strand to the non-template strand, compensating the melting of the dsRNA product, leading to the backtracking of the RdRp. At high force (which results in a larger distance between single nucleotides on the non-template strand), the increase in tension impairs the rebridization of the template to the non-template strand, preventing backtracking to happen.

To examine the force dependence of backtracking, we acquire data at forces of 16 pN, 20 pN, 25 pN, 30 pN and 35 pN (blue, cyan, green, yellow and red respectively, Figure 2A) using the buffer conditions described in Materials and Methods. In this range, the applied force destabilizes the ds-ssRNA junction by $0.6 - 2k_B T$ (8). We perform a dwell-time analysis for each force (Figure 2A,2B) and find that the recorded dwell-times span almost five orders of magnitude in time. Each distribution contains between ≈12000 (35 pN) and ≈30000 (20 pN) dwell-times. From Figure 2B, we observe that the long-lived pauses representing the backtracked state (Figure 1D) are populated to a degree that depends on the applied force: higher force provides lower probability density distribution of the dwell-times. We observe a force dependent hierarchy from 16 pN to 35 pN, with the error bars (one standard deviation confidence interval extracted from 1000 bootstrapped data sets) of the different distributions being well separated. The probability density distribution of the dwell-times does not depend on the presence of P2 RdRp in the reaction buffer, as shown in a previous study (Figure S5 of (8)). In the same study, we also observed that the lifetime of the exponentially distributed pauses (Figure 1D) does not exceed 17 s at [NTP]_opt_ (8), and to avoid the influence of shorter non-backtracked pauses (region (ii) in Figure 1D), we similarly score only pauses longer than 20 s as in (8). In this way, we measure the probability of P2 to pause longer than 20 s within 10 nt incorporation for each force (Figure 2C). We observe a strong force dependence, where for example the probability of being in a pause longer than 20 s during a 10 nt incorporation cycle is 0.0228 ± 0.0012 at 16 pN and decreases nearly 20-fold to 0.0013 ± 0.0003 at 35 pN (Figure 2C).

### The P2 RdRp backtracked state is influenced by template strand rehybridization

The force dependence of the backtracking behavior of P2 RdRp can be understood in terms of the model illustrated in Figure 2D. In this model, thermally induced fraying of the duplex region formed between the newly synthesized and the template strand will liberate the 3′-end of the nascent RNA strand. Further exposure of the nascent RNA strand, and concomitant backtracking by P2, is facilitated by the rehybridization of the downstream template strand with the 4.2 kb (+) strand. Tension in the template strand lowers the energetic gain of hybridization with the plus strand, and thus the rehybridization process is favored at lower forces (34) (Figure 2D). Provided that the exposed 3′-end can be accommodated (the NTP channel being a likely candidate, see e.g. PDB 1UVI (35)), backward diffusion of P2 RdRp along the template strand will be favored at lower forces, consistent with the observed increased probability for long backtracked pauses (Figure 2C). As an applied force changes the energy gain for hybridization between the tether and plus strand, our model suggests a possible sequence dependence of the backtracked state as determined by base-pairing energies.

### P2 RdRp reverses direction during transcription

**Figure 3.**
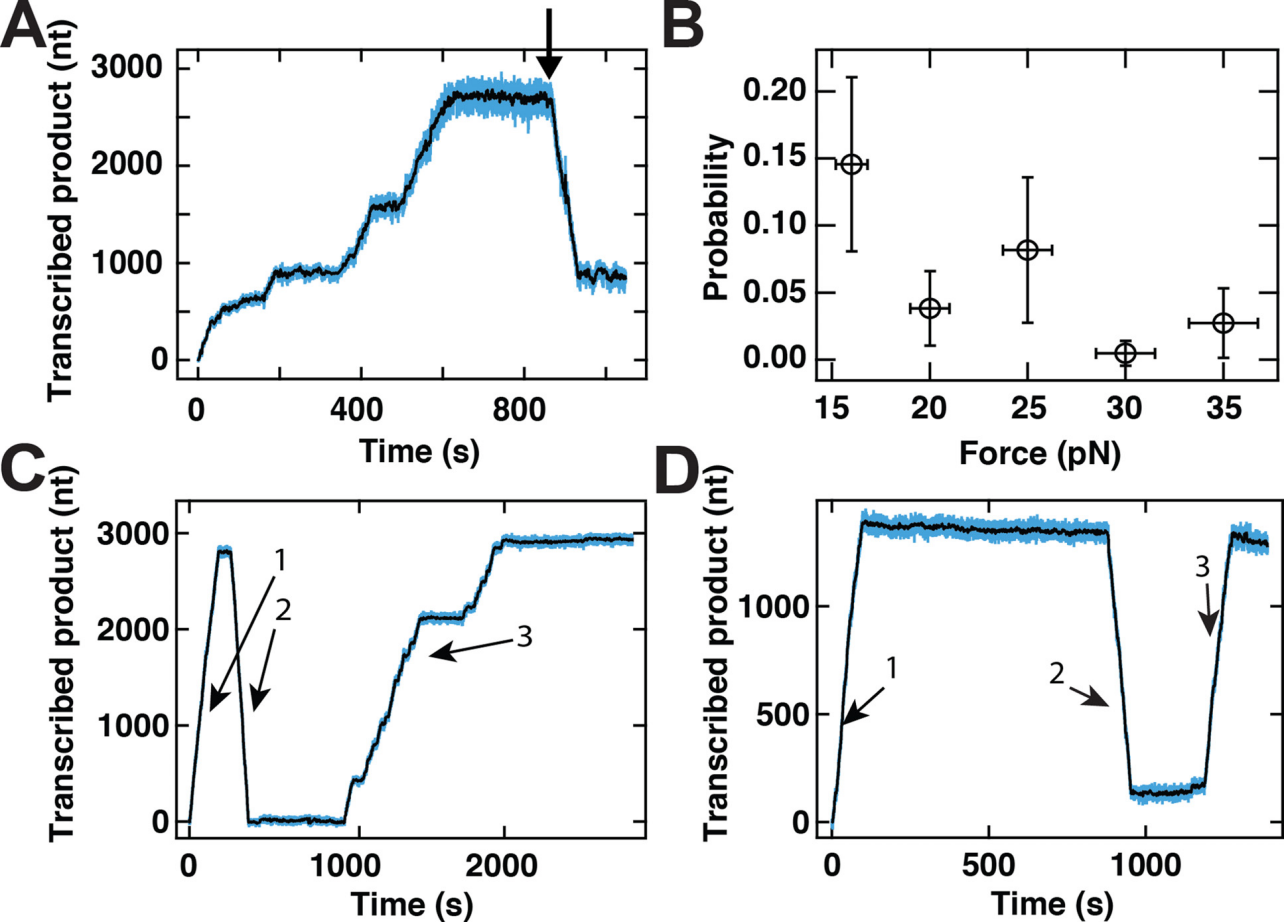
Long pauses in the elongation dynamics are rarely followed by processive changes in the direction of transcription. **(A)** The extension of the transcribed product as a function of time. At 800 s (indicated by arrow), P2 RdRp exhibits a reversal behavior. The experimental conditions include an applied force of 16 pN force and an acquisition frequency of 25 Hz. **(B)** The probability of observing a reversal event as a function of the applied force (see panel (A)). The error bars represent the 95% confidence interval for a binomial distribution for the ordinate and the standard deviation of the applied force (±5%, see (50)) for the abscissa. **(C** and **D)** P2 RdRp transcription activity traces that exhibit very rare behavior in which multiple switches in the apparent directionality of the signal can be observed. In both panels, the extension of the RNA construct (1) increases, then (2) decreases (as in a reversal event) and (3) increases again. Data are acquired at an applied force of 25 pN using buffer conditions described in Materials and Methods. For panels (A), (C) and (D), raw data are shown in blue and data low-pass filtered at 0.5 Hz are shown in black.

In our single-molecule experiments, we occasionally observe events in which P2 appears to ‘reverse’ its direction during transcription (Figure 3A). Prior to such events, P2 transcript elongation starts normally (Figure 3A, data up to ≈600 s). However, following a long pause, we occasionally observe a decrease in the extension of the RNA tether (Figure 3A, black arrow). This decrease typically continues until the tether length of the RNA construct reverts to its original value measured prior to P2 initiation. Such ‘reversal’ events occur in a small percentage of all traces. The occurrence of reversals in a trace is, however, significantly more likely at applied forces below 20 pN (Figure 3B), with a probability of 0.15 ± 0.07 and 0.027 ± 0.026 at applied forces of 16 pN and 35 pN, respectively. In very rare instances (0.2% of P2 traces collected), we also observe a renewed ‘forward’ motion upon the completion of a reversal (Figure 3C,3D).

To understand the origin of the reversals, we perform two additional experiments. First, we test whether the reversal events could be attributed to polymerization by P2 on the single-stranded regions exposed on the RNA tether as transcription progresses. We test this by monitoring the extension of an RNA construct that lacks the template strand (Figure 1A) in the presence of P2 at 16 pN applied force. From the observation of 46 tethers, no active polymerization events are detected. Second, we test whether the reversals require the presence of free P2 in the flow cell buffer. To this end, we stall P2 on the template strand by depriving it of one NTP, rinse the flow cell and flush in the reaction buffer containing all four NTPs but devoid of free P2 (8). Under these conditions, 50 data traces displayed continued transcription by P2, yet not one of these contained a reversal, further demonstrating the need of at least a second RdRp for reversal to occur. As the probability of observing a reversal event at this force (16 pN) in the presence of free P2 is 0.15 (Figure 3B), the probability of not observing a single reversal in 50 traces can be estimated to be less than 0.0003.

**Figure 4.**
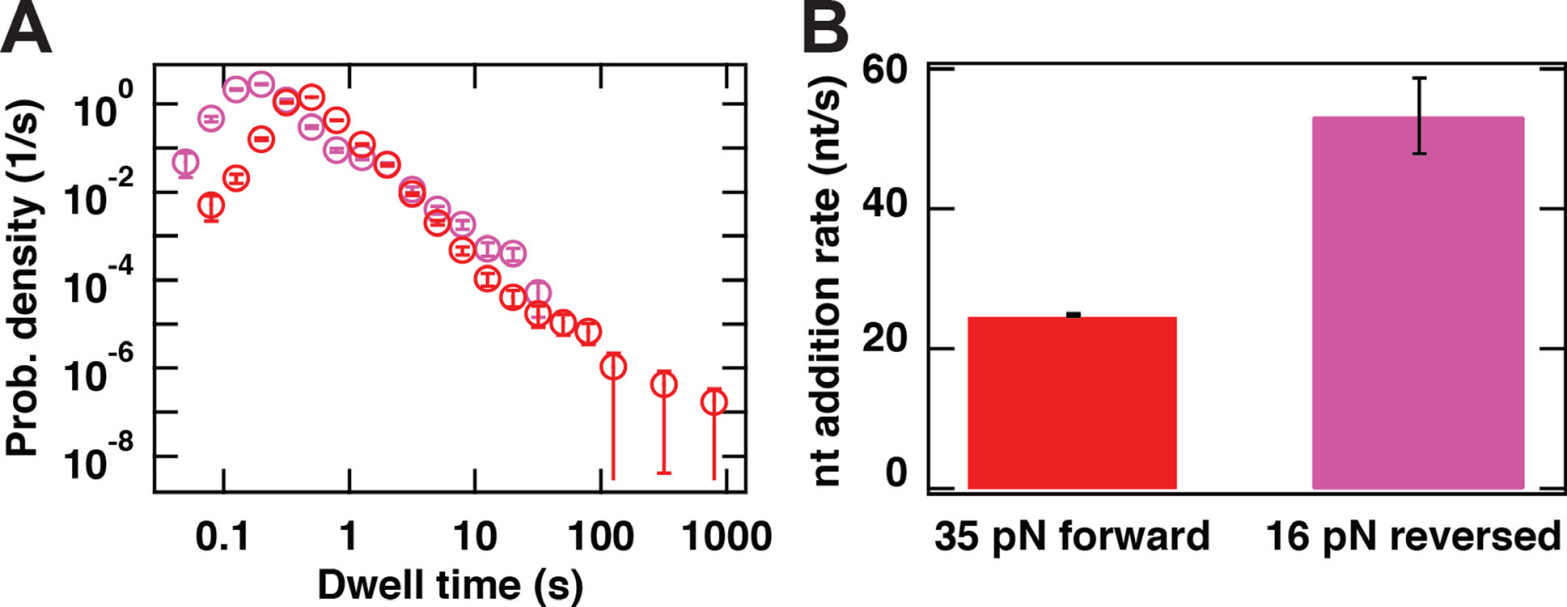
P2 RdRp exhibits different kinetic properties in standard elongation compared to reversal events. **(A)** The dwell-time distribution for 16 reversal traces obtained at 16 pN (pink circles) and for the forward traces obtained at 35 pN (red circles). Error bars are one standard deviation confidence intervals derived from 1000 bootstraps. **(B)** The nucleotide addition rates extracted from the maxima of the dwell-time distributions in (A) (identical color codes employed). Error bars are one standard deviation confidence intervals derived from 10000 bootstraps.

Lastly, reversals appear to be associated with a higher rate of nucleotide addition and a decrease in pauses frequency in comparison to initial forward transcription (Figure 3A). To quantify this, we utilized the largest data set of observed reversals (acquired at 16 pN) to construct a dwell-time distribution derived only from the reversed part of the traces (pink circles, Figure 4A). We can determine the maximum of the distribution, which reports on the apparent catalytic rate. Notwithstanding the lower statistics for the reversals data set at 16 pN, it is clear that the peak position shifts to shorter time in comparison with the peak position of the data set at 35 pN (red circles, Figure 4A), the largest assisting force. The corresponding apparent nucleotide addition rate can be determined as 53.4 ± 5.4 nt/s (Figure 4A,4B). This is nearly 2-fold higher than the rate of nucleotide addition extracted from the forward transcriptions at the same NTP concentration and 35 pN (25.6 ± 0.2 nt/s) (Figure 4B) (8).

## DISCUSSION

RdRp is involved in the replication and the transcription of the viral genome and is therefore essential for virus survival. While the catalytic activity of RdRps has been intensively studied in bulk (36–38) and, more recently, in single-molecule experiments (8), non-catalytic pauses such as backtracked pauses have not previously been directly observed in RdRp elongation dynamics. For the first time, we show direct evidence for the backward motion characteristic of backtracking (Figure 1C) for a viral RdRp. The statistics provided by our experimental assay have been key in quantifying the long-lived pauses that result from a polymerase backtracking. Considering pause dynamics over more than two orders of magnitude in time, we show how the energy needed to propagate the fork influences the probability of backtracking.

**Figure 5.**
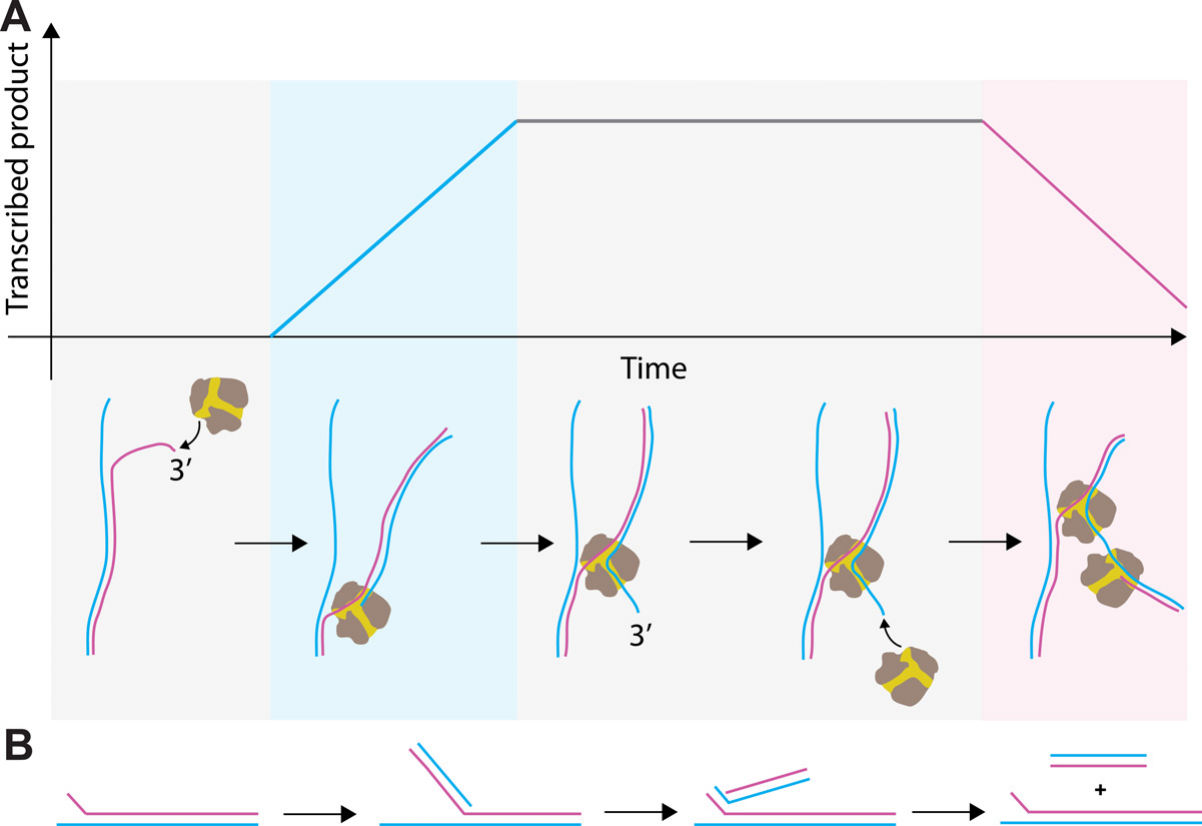
Mechanism accounting for the observation of P2 RdRp reversal events. **(A)** (Top) The mechanochemical model and (bottom) a schematic trace for a reversal event (Figure 3A). Reversal events are proposed to originate from a second P2 RdRp that initiates on the 3′ RNA product end that extrudes from the NTP channel of a first, backtracked P2 RdRp. The second P2 RdRp is free to initiate template-dependent RNA polymerization on the exposed RNA 3′-terminus. Subsequent elongation will cause the first P2 RdRp to be pushed back along the template strand. (+)RNA is shown in blue and (−)RNA is shown in pink. The background shading represents the different stages of RNA synthesis: gray for the absence of catalytic activity, blue for (+)RNA synthesis and pink for (−)RNA synthesis. **(B)** Schematic representing RNA production first by the forward transcribing RdRp and then by the second RdRp upon reversal (color code identical to (A)). Strands aligned in parallel fashion are fully hybridized. The net product of a complete reversal event is the original dsRNA construct plus a shorter dsRNA.

The proposed model for backtracking (Figure 2D) also accounts for the observed reversals. Backtracking can introduce a newly exposed 3′-end for a new polymerase to initiate on, and we hence interpret the reversals as a sign that a new polymerase has initiated and drives the ssRNA/dsRNA junction backward (Figure 5A). This association between backtracks and reversal events is supported both by the similar force dependence of their probabilities (compare Figures 2C and 3B) and the fact that free P2 in solution is required for the observation of reversals.

Alternative scenarios that are consistent with the observed decrease in extension can be eliminated. These include exonuclease activity by P2, as this is not supported by structural evidence (22); and nucleotide excision by P2 via pyrophosphorolysis, as results on the structurally-related bovine viral diarrhea virus RdRp (39) and human immunodeficiency virus (HIV) reverse transcriptase (40) indicate that pyrophosphate concentrations as high as 100 μM would be required, and these are neither present in our fresh HPLC-purified NTP stock nor produced by the polymerization activity in our single-molecule assay given the low number of active elongation complexes. A recent study has shown that the hepatitis C virus RdRp is able to use an NTP to excise the nucleotide at the 3′ end (38). However, the catalytic rate observed for such a reaction is three orders of magnitude slower than what the catalytic rate we measured during a reversal with P2 RdRp (Figure 3B). Furthermore, this nucleotide excision mechanism has only been demonstrated with a C terminated 3′ RNA product (38), whereas our template does not contain long stretches of C residues. We can also discard a scenario in which dissociation by an initial P2 from its template strand is followed by subsequent initiation of a second P2 on the exposed 3′ end of the newly synthesized complement. While such a scenario would account for the presence of a second P2, it contradicts observations that P2 forms a stable elongation complex (as demonstrated in our previous work in which we are able to reactivate P2 following a significant period of stalling (8)) and fails to explain the observed force-dependence of the probability of reversals (Figure 3B). It remains intriguing that the nucleotide addition rate is found to be 2-fold higher during reversals than during forward transcription activity (Figure 4A,4B). Two P2 enzymes working in close proximity thus seem to favor a more rapid translocation, but elucidating the reasons behind this will require further in-depth study. Interestingly, from the dwell-time distribution of the reversals at 16 pN applied force (Figure 4A), we observe that the second polymerase does not enter into long pauses. One can explain this observation using the backtrack model of Figure 2D: at low applied force, the small energy difference between RNA tether-RNA template base pairs and the RNA product-RNA template base pairs facilitates the re-hydridization of the RNA template strand to the RNA tether. This favored hybridization prevents backtracking of the second enzyme, in a manner similar to that of the first enzyme in the high force situation.

### Implications of our model

A link can be made between the observed force dependence of backtracking and the behavior of P2 within the Φ6 bacteriophage. Within the bacteriophage, the hexameric NTPase (denoted P4) operates as a conduit for the plus-strand RNA release (41–43). By analogy with the role of the applied force in our magnetic tweezers experiments, we speculate that P4 may, by limiting the excursions of the plus-strand RNA, prevent P2 from becoming trapped in a backtracked state. Its anchoring capability could, amongst its other known roles (41), serve to maintain the overall P2 transcription rate and directionality. However, we cannot exclude the occurrence of backtracking within the viral capsid. Furthermore, given the presence of ≈10 P2 RdRps in the viral capsid (44), a number of them may be free of template (the viral genome being tri-segmented (45)). Occurrences of P2 RdRp backtracking under these conditions allow for the possibility of reversal events to occur within the viral capsid.

The biological implications of the ability of a second P2 RdRp being able to initiate *de novo* on an exposed 3′-end remains to be further investigated. A possible role for such reversal events could be the production of shorter RNA molecules (Figure 5B) that would be subsequently used for homologous (46) or heterologous recombination (47–49). Indeed, it has been shown that two genomic segments of Φ6 can recombine to replace the damaged 3′-end of one of the segments (48). One might speculate that the second polymerase could rescue the template on which the first one is stalled, either for intrinsic biochemical reasons (e.g. catalytically inactive) or due to the presence of a roadblock on the dsRNA. The nearly 2-fold higher rate of reversal events compared to forward transcription events could potentially be exploited by the virus to enhance the production of viral RNA in the host cell. Another interesting observation is that the sequence of events shown in Figures 3D and 5A would result in the amplification of a viral RNA that contains an incomplete part of the genomic template (Figure 5B). Future work will be necessary to unravel any sequence dependence of the reversals. The degree of structural similarity between RdRps (18) suggests that this mechanism could be conserved amongst RNA viruses that initiate replication or transcription *de novo* (e.g. hepatitis C virus (26)).
